# MD-TSPC4: Computational Method for Predicting the Thermal Stability of I-Motif

**DOI:** 10.3390/ijms22010061

**Published:** 2020-12-23

**Authors:** Amen Shamim, Maria Razzaq, Kyeong Kyu Kim

**Affiliations:** 1 Department of Precision Medicine, Institute for Antimicrobial Resistance Research and Therapeutics, Sungkyunkwan University School of Medicine, Suwon 16419, Korea; amenshamim@gmail.com (A.S.); mariarazzaqtoor@gmail.com (M.R.); 2 Center of Agricultural Biochemistry and Biotechnology (CABB), University of Agriculture, Faisalabad 38040, Pakistan

**Keywords:** i-motifs, molecular dynamics simulation, melting temperature, CD spectroscopy, thermal stability

## Abstract

I-Motif is a tetrameric cytosine-rich DNA structure with hemi-protonated cytosine: cytosine base pairs. Recent evidence showed that i-motif structures in human cells play regulatory roles in the genome. Therefore, characterization of novel i-motifs and investigation of their functional implication are urgently needed for comprehensive understanding of their roles in gene regulation. However, considering the complications of experimental investigation of i-motifs and the large number of putative i-motifs in the genome, development of an in silico tool for the characterization of i-motifs in the high throughput scale is necessary. We developed a novel computation method, MD-TSPC4, to predict the thermal stability of i-motifs based on molecular modeling and molecular dynamic simulation. By assuming that the flexibility of loops in i-motifs correlated with thermal stability within certain temperature ranges, we evaluated the correlation between the root mean square deviations (RMSDs) of model structures and the thermal stability as the experimentally obtained melting temperature (Tm). Based on this correlation, we propose an equation for Tm prediction from RMSD. We expect this method can be useful for estimating the overall structure and stability of putative i-motifs in the genome, which can be a starting point of further structural and functional studies of i-motifs.

## 1. Introduction

Genomic DNA can adopt various conformations including the canonical right-handed B-form and non-B form DNA or the noncanonical DNA (ncDNA) including guanine-quadruplexes (G4), cytosine-quadruplexes (C4), triplexes, Z-DNA, cruciform and hairpins [1,2,3,4,5,6]. The ncDNAs are formed in repeated sequences in the genome and participate in various biological functions such as controlling replication, maintaining genome integrity and regulating transcription and translation activities [7,8]. Considering the imperative roles of the ncDNA structures in regulating many genetic events and genome integrity, it is necessary to identify ncDNAs with repeated sequences and investigate their structure and functions for comprehensive understanding of the genome. 

Among ncDNAs, G4s have been most widely investigated since they have been reported in telomeres, where they affect the binding of certain proteins to maintain telomere integrity during meiosis [1]. The significant prevalence of G4 in human gene promoters, 5′ and 3′ untranslated regions [9,10] and even coding regions [11,12] indicates that G4s have a role in gene regulation. G4 formation is also implicated in diseases including cancer. Several oncogenes such as *c-MYC, VEGF, PARP-1, BCL2* and *K-RAS* [13,14,15,16,17] have been identified to be regulated by G4s, since their expression is controlled by G4 formation. The genome-wide identification and characterization of G4s in various organisms have been reported in experimental and computational approaches [18,19,20,21,22]. These findings have shown that G4s are present in various locations in the genome but are enriched in gene promoters in human and mouse genomes compared with other non-mammal species [23].

Intercalated motifs (I-Motifs), also called as cytosine-quadruplexes (C4, can be formed on the complementary strand of G4 in a mutually exclusive manner [24]. The i-motif consists of two parallel duplexes stabilized by hemi-protonated cytosine-cytosine base pairs that intercalate into each other in an antiparallel manner [2]. The i-motif is involved in biological processes, as the transcription of genes can be regulated by the formation or destabilization of i-motif structures, especially in non-coding regions [25,26]. In addition, the structural change in DNA caused by the i-motif causes replication errors that can enhance cancer progression [8]. Since the i-motif presents complementary to G4s, i-motifs are also expected to be involved in cancers. Indeed, i-motif structures have been reported in the promoters of several oncogenes, including *BCL2*, *c-KIT*, *c-MYC*, *VEGF*, *RET*, *RB*, and *ILPR* [14,24,27,28,29,30]. Recent studies provided cellular evidence supporting the presence of i-motifs at regulatory regions in the genome of human cells and their roles in gene regulation [31]. These findings have suggested the i-motif as a potential therapeutic target in cancer. Considering the number of G4s in the genome, numerous i-motifs are expected to be present and play essential cellular roles. Therefore, the biophysical and functional characterization of i-motifs is urgently required. However, so far, only a few structural and biophysical studies of i-motifs have been carried out, possibly due to the dynamic nature of i-motifs and time required for structural studies of individual i-motifs.

The stability of the i-motif is correlated with its functionality, since its regulatory function can be increased when its stability is enhanced by chemicals or structural alteration [32]. Accordingly, it is necessary to determine the structure and stability of an i-motif to estimate the functional significance or relevance to a disease with any known i-motifs. The stability of an i-motif depends on many external factors such as pH, ionic strength, temperature. The most important factor is the characteristics of the i-motif sequence, especially the length and base in the loop since the core C:C base pair structure is well conserved [33,34]. However, it is also suggested that a cytosine modification might have different effects on i-motif stability depending on the environmental conditions [35]. Considering the number of i-motifs and the limitation of experimental tools for structural studies, an alternative approach to predict the structure and stability of i-motifs is highly necessary. To provide one possible solution to this issue, we developed a novel computation method, MD-TSPC4, to predict the thermal stability of i-motifs based on molecular modeling and molecular dynamic (MD) simulation. We confirmed that the thermal stability of uncharacterized i-motifs was well predicted when comparing with the Tm value obtained by the experimental method. Therefore, we anticipate that this method can be widely used for estimating the stability of putative i-motifs in the genome, which can be a starting point for further structural and functional studies of i-motifs. 

## 2. Results

### 2.1. Research Hypothesis and Molecular Modeling

In many cases, structural stability directly contributes to the biochemical activity of ncDNA structures. For example, the inhibitory activity of i-motifs and G4 on transcription is enhanced when their structures are stabilized [29,36]. Therefore, it is important to investigate the structural stability of i-motifs to understand their biochemical and biological relevance. Structural stability can be interpreted as the thermal stability by experimental analysis. However, considering the number of i-motifs present in the human genome and limited accessibility of experimental tools, systematic investigations of structural stability of i-motif have been limited. Therefore, a computational method to predict the thermal stability of the i-motif is necessary for high throughput analysis of key i-motif structures in the genome. Several studies reported that the i-motif loop length is an important contributing factor for thermal and pH stability [34]. In this study, we anticipated developing a novel computational method to predict the thermal stability of the i-motif based on the hypothesis that the loop flexibility of the i-motif is highly correlated to the thermal stability. For this purpose, we built several model i-motifs with different loop lengths and investigated the coefficient of determination (R2) between the thermal stability determined by experimental methods and loop flexibility estimated by MD simulation using model i-motifs. Finally, we propose a new computation method for the prediction of i-motif stability based on this correlation.

In this study, we hypothesized that the loop region may be the most important factor affecting the structural stability of i-motifs, while the core structure with cytosine repeats is well conserved. Since a previous study reported that thymine in the loop region is highly responsible for thermal stability [37], we examined the effect of thymine bases on the structural stability. Based on this hypothesis, we designed three i-motif models containing loops with different lengths (Table 1), short (TTT), medium (TTTTT) and long (TTTTTTT) loops, and named them as CCCT3, CCCT5 and CCCT7, respectively. The three intramolecular loops connecting four cytosine repeats were named loop1, loop2 and loop3 (Table 1), in order. The crystal structure (PDB ID: 1ELN) of the human telomeric i-motif with the sequence of d[(CCCTAA)3CCCT] was used as a template for modeling. While the core structure formed by cytosine repeats was fixed, the “TAA” in the 1ELN structure was replaced by extended loops, T3, T5 and T7, for the loop modeling. Modeled structures were further refined by energy minimization to remove the steric clashes between the atoms.

### 2.2. MD Simulation and Trajectory Analysis

For MD simulation, we added partial charges to the i-motif models for maintaining protonated C-C+ base pairing. We performed MD simulation calculations for 200 ns at different thermal conditions, 280 K, 300 K, 320 K, 340 K, and 360 K, to estimate the dynamic behaviors within a 0 to 100 °C range. All calculations were observed under explicit solvent at neutral pH. Since our initial models are manually built rather than using an experimental crystal structure, we began heating for a very short time to relax the initial models and then gradually increased the time step in equilibration. Initially, we applied weak restraint by keeping the i-motif fixed to control the heating. In equilibration, we removed the restraints and started the time step of i-motif at 300 K (room temperature). We applied these criteria individually on all the models for simulating the lower and elevated temperatures. 

**Figure 1:**
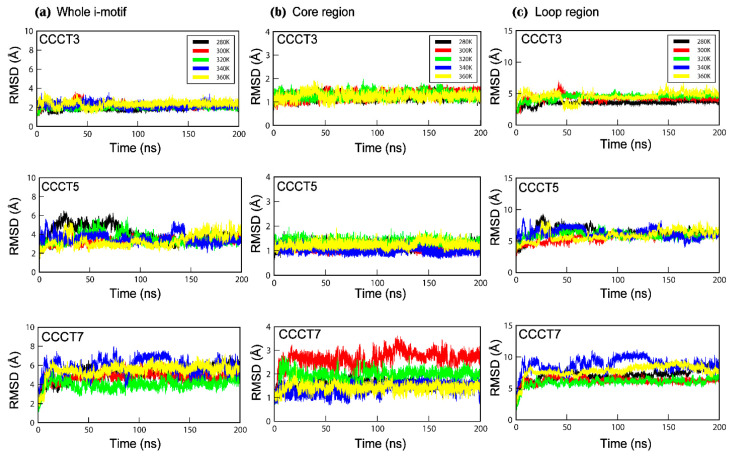
The root mean square deviation (RMSD) for hemi-protonated i-motif structures, CCCT3, CCCT5 and CCCT7 during 200 ns MD simulations under different thermal conditions; 280 K (black), 300 K (red), 320 K (green), 340 K (blue), 360 K (yellow). The first frame in each i-motif trajectory was used as the reference point for RMSD calculations. RMSD changes of atoms in the whole i-motif (**a**), the core region (**b**), and the loop region (**c**) are presented.

We extracted the trajectory from i-motif models with short (CCCT3), medium (CCCT5) and long (CCCT7) loops at each temperature, and the structural fluctuation was displayed by RMSD of atoms in the whole i-motif, the core region and in the loop region (Figure 1). MD simulation analysis revealed that the structural stability of the i-motif, represented by the overall RMSD change, is inversely proportional to the loop length, since RMSDs for 200 ns at near room temperature (300 K) were 1.9 Å, 3.3 Å and 4.6 Å for CCCT3, CCCT5 and CCCT7, respectively (Figure 1a and Table 2). We anticipated that the structural change would increase at high temperature. Unexpectedly, the overall RMSD was not proportional to the temperature in the case of CCCT5 and CCCT7 while it was almost linearly proportional to the temperature in CCCT3 (Figure 1 and Table 2). 

**Figure 2:**
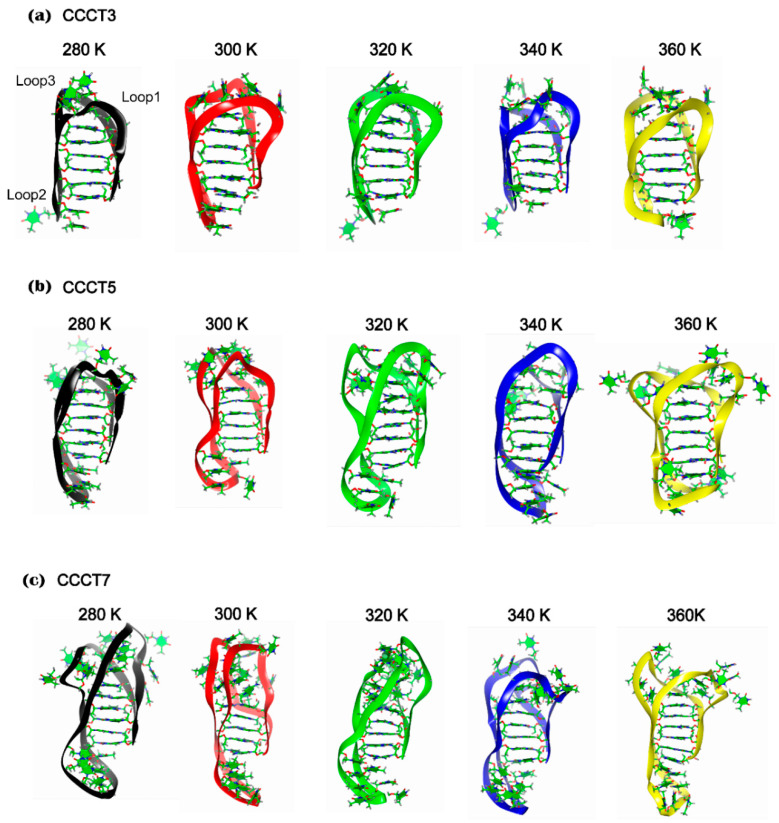
Three-dimensional representation of model i-motifs, CCCT3 (**a**), CCCT5 (**b**) and CCCT7 (**c**), in production run 200 ns MD simulation at five different temperatures; 280 K (black), 300 K (red), 320 K (green), 340 K (blue), 360 K (yellow). The backbone and bases are represented in ribbon and stick diagrams, respectively. Three intramolecular loops, loop1, loop2 and loop3, are indicated in the CCCT3 model (280 K).

Higher RMSD values in the model i-motifs are mostly because of the structural flexibility of the loops rather than that of the core region since RMSD of the loop region showed much deviation for 200 ns simulation in comparison to that of core region (Figure 1b,c and Table 2). This analysis is also well supported by the conformational diversity of the loop region of each model obtained at 200 ns simulation (Figure 2 and Figure S1) in which loop regions are highly deviated from the core region when the loop length is long. In addition, regardless of temperatures, it is also observed that the RMSD values in the loop region are proportional to the length of the loop (Figure 1c and Table 2). Therefore, these findings indicate that the length of the loop connecting with the cytosine core region affects the structural stability of the i-motif, which are consistent with previous experimental data [37].

While loop region showed the high conformational flexibility as represented by the high RMSD, the overall structure of the core region seems to be well maintained as RMSD is less than 2.5 Å regardless of loop length and temperature (Figure 1b and Table 2) (except CCCT7 at 300 K). Therefore, the structural integrity of i-motifs seems to be well maintained by the structural stability of the core region. The most important factor affecting the structural stability of the core region is hydrogen bonds between the neutral cytosine and protonated cytosine (C:C+) since the distances between the two CN3 in the protonated i-motif structures were well maintained within hydrogen bond distance throughout the simulation (Table S1). This interpretation is also supported by the analyses of the radial distribution function (RDF) for hydrogen bonding between cytosine bases in the core region (Figure S2). The radial distribution functions (RDF), symbolized as g(r), of the initial (1 ns) and final models (200 ns) revealed the sharp peaks near distance of 2.0 Å regardless of temperature and loop length, suggesting that C:C+ hydrogen bonds in the core region are stably maintained during simulation (Figure S2). Exceptionally, in the case of CCCT7, the cytosine base pair (C2c:C4a) is disrupted during simulation at 300 K (Figure 2c and Table 2), which results in the higher RMSD in the core region. When the simulation was performed without protonation on cytosine, the RMSD of core region was highly increased depending on simulation time and the overall structure disintegrated (Figure S3). These simulation analyses of the core region are well consistent with the previous experimental evidence indicating that base pairs between the protonated cytosine in the i-motif play a key role in stabilizing the structure [38]. 

### 2.3. Conformational Flexibility 

**Figure 3:**
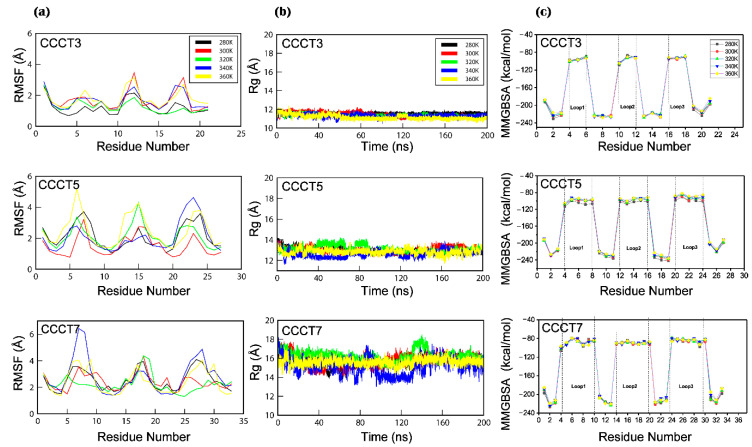
Conformal flexibility of the model i-motifs. (**a**) The per residue root mean square fluctuation (RMSF) of each residue of the model i-motif structures, CCCT3, CCCT5 and CCCT7, in production run of 200 ns MD simulation are displayed. (**b**) Changes of the average radius of gyration (Rg) for the hemi-protonated C:C+ base-pairing are displayed during 200 ns molecular dynamics simulation. (**c**) Molecular Mechanics Generalized Born Surface Area (MMGBSA) binding free energy are plotted per residue. RMSF, Rg and MMGBSA values of model structures obtained during the MD simulation under five thermal conditions, 280 K (black), 300 K (red), 320 K (green), 340 K (blue), and 360 K (yellow) are displayed in different colors.

The conformational flexibility of each region of i-motif was analyzed by the RMSF analysis of the model obtained after 200 ns MD simulation (Figure 2 and Figure 3a). Consistent with the trajectory analysis, in RMSF, the protonated CCCT3 i-motif structure shows less fluctuations compared with the CCCT5 and CCCT7 structures (Figure 3a). In structural analysis, we learned that the structural fluctuations are largely caused by the flexible movement of the loop region since the cytosine core region in all structures at all temperatures remains stable whereas the loop region undergoes flexible movement (Figure 2). Since it has been reported that interaction between loop1 and loop3 contributes to structural stability of d[(CCC)3TAACCC] [39], high loop flexibility of medium (CCCT5) and long loop (CCCT7) i-motifs is likely to be caused by the loosing loop-to-loop interaction. Indeed, in the current simulation model, we observed that the loop interaction is not present in CCCT5 and CCCT7, but present in CCCT3 (Figure S1). Another indicator of structure stability is the radius of gyration (*Rg*) that describes the overall shape and compactness of the i-motif structure. The average values of radius of gyration for the short (CCCT3) and long loop (CCCT7) structures during 200 ns measured 11.1 Å and 15.4 Å, respectively, indicating that the overall protonated CCCT3 system remains stable throughout the simulation time (Figure 3b). 

We also examined the contribution of the structural stability of the i-motif by calculating the binding free energy of each residue (Figure 3c). The per residue (decomposition) analysis revealed that the loop regions of all the i-motifs structures showed much higher energy than the core region. The base pairing interactions between the C:C+ i-motif core region is responsible for the smaller energy change which maintains the neighboring interactions among the bases and stabilizes the i-motif core region. 

### 2.4. Experimental Measurement of Thermal Stability

**Figure 4:**
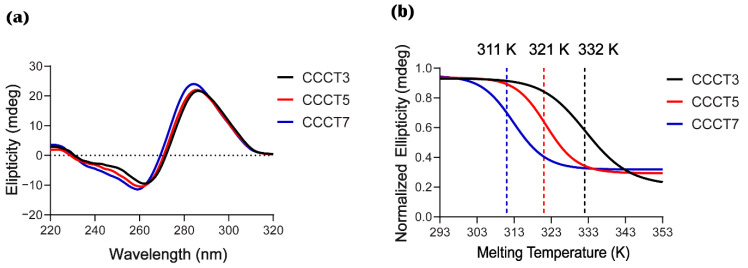
CD analysis of ODNs of the model i-motifs (CCCT3, CCCT5 and CCCT7). (**a**) CD spectra of i-motif oligos at the concentration of 15 mM. Each spectrum is an average of 3 measurements in the wavelength range between 220–320 nm. (**b**) The thermal melting analysis of i-motif oligos at the concentration of 15 µM annealed in 20 mM sodium cacodylate buffer at pH 5.0. CD melting graph was obtained at 285 nm wavelength within temperature range (293–353 K). Three independent measurements were performed. An average Tm value for each i-motif sample is shown in the graph.

Our MD simulation results demonstrated that a short loop structure has a more stable and compacted structure compared with loop long i-motif structures (Figure 2, Figure S1). Since the structural stability is correlated to the thermal stability, we experimentally examined the thermal stability of oligonucleotides (ODNs) of the model i-motifs using CD, which is commonly used in biophysical studies for detecting the formation of i-motif structures and thermal stability. The typical CD spectrum of i-motif at pH 5.0 shows a positive and negative signal near 285 and 265 nm, respectively, with the ellipticity of peak at 285 nm that is two times higher than the peak at 265 nm [40]. Consistent with these results, three model i-motifs showed spectra similar to the typical i-motif, suggesting that model i-motifs form an i-motif structure (Figure 4a). The melting temperature (Tm) and annealing temperature (Ta) of each model i-motif were measured to evaluate their thermal stability by monitoring ellipticity change at 285 nm. ODNs with a shorter loop (CCCT3) exhibited the highest Tm value (332 K) while CCCT5 and CCCT7 followed a linear trend with decreasing values of 321 K and 311 K, respectively (Figure 4b, and Figure S4). 

### 2.5. Correlation between Thermal Stability and Conformational Flexibility 

**Figure 5:**
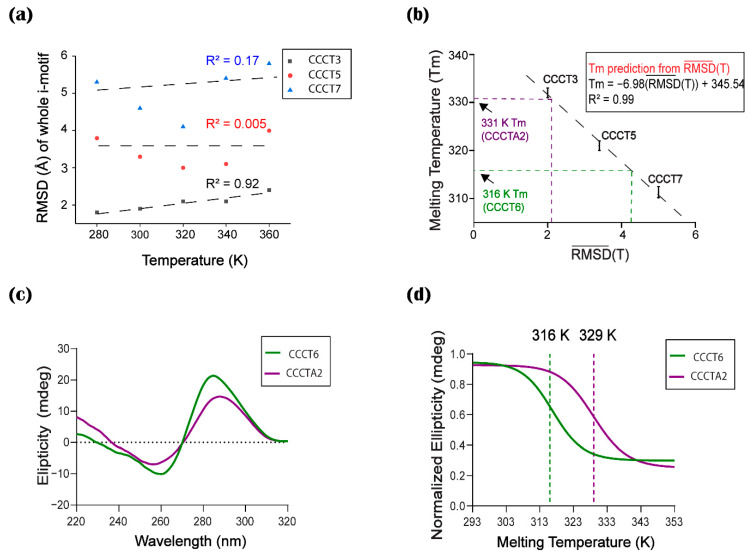
Correlation between Tm and RMSD. (**a**) The correlation between the root mean square deviation (RMSD) of whole atoms in the i-motif CCCT3 (black), CCCT5 (red) and CCCT7 (blue) for 200 ns simulation at five temperatures (280, 300, 320, 340, and 360 K) and each corresponding temperature are plotted with the coefficient of determinant (R2). (**b**) The correlation between the $\bar{RMSD}\left(T\right)$ and experimental Tm of model i-motifs (CCCT3, CCCT5, and CCCT7) is drawn as a TR plot (black dotted line). The correlation of determinant (R2) is 0.99. Based on this TR plot, Tm value is obtained by the linear regression equation, Tm = −6.98 × $\bar{RMSD}\left(T\right)$ + 345.54. The predicted Tm values of model i-motif CCCT6 (green) and CCCTA2(magenta) are indicated in the TR plot. (**c**) Experimentally determined CD spectra of CCCT6 and CCCTA2. (**d**) CD melting temperature (Tm) of CCCT6 and CCCTA2 determined from the CD thermal melting analysis at 285 nm. Experimental conditions for CD experiments are depicted in Figure 4.

We hypothesized that thermal stability can be estimated by plotting the RMSD vs. temperature in the similar way as determining the Tm values by CD experiments based on the assumption of the correlation coefficient between temperature and conformational flexibility represented by RMSD in MD simulation. However, the RMSD vs. temperature plot for whole i-motif (Figure 5a) revealed that only CCCT3 has a correlation between RMSD and temperature. This result suggests that the structural alteration of the long loop might not only be affected by temperature but also other unknown factors under these simulation conditions. The correlation in the RMSD vs. temperature plot (Figure 5a) revealed that only CCCT3 has a linear correlation between RMSD and temperature. However, due to the lacking correlation between the RMSD and temperature in the long loop i-motifs (Figure 5a), this method cannot be applied to estimate the Tm value for i-motifs. Interestingly, we noticed that the average RMSD value for the five temperature range ($\bar{RMSD}\left(T\right)$) is proportional to the loop length; the $\bar{RMSD}\left(T\right)$ of all atoms in CCCT3, CCCT5 and CCCT7 for 200 ns simulation under five thermal conditions are 2.0 Å, 3.4 Å and 5.0 Å, respectively (Table 2), confirming that the i-motif with a short loop is less flexible or more stable than the i-motif with long loops. However, when $\bar{RMSD}\left(T\right)$s were calculated using either core or loop atoms, the correlation between $\bar{RMSD}\left(T\right)$ and Tm values was not high (Table 2). Therefore, we plotted the $\bar{RMSD}\left(T\right)$ values of all atoms obtained by MD simulation for five temperatures against the experimentally determined Tm values (Figure 5b). This Tm vs. $\bar{RMSD}\left(T\right)$ plot (TR plot) showed a high correlation between two values with a correlation coefficient of 0.99. The resultant plots acquired the linear trend in all testing sets. Therefore, our results confirmed that the structural stability of i-motifs estimated by MD simulation was in good agreement with experimental results regardless of the loop length when the average RMSD was used for comparison.

### 2.6. Validation of the Computational Prediction

To further test if the TR plot can be used for estimating the thermal stability of any unknown i-motif, we introduced two additional i-motifs: CCCT6, an i-motif with T6 in all three loops, and CCCTA2, an i-motif found in human telomeres with a TAA loop [39]. In the same way as the previous model i-motifs, the $\bar{RMSD}\left(T\right)$s of CCCT6 and CCCTA2 were calculated based on the MD simulation for 200 ns at five different temperatures. The $\bar{RMSD}\left(T\right)$s of CCCT6 and CCCTA2 are 4.1 Å and 2.1 Å, respectively (Table 2). By applying these values on the TR plot, Tm values were estimated to be 316 K and 331 K for CCCT6 and CCCTA2, respectively (Figure 5b). We then measured the Tm values by experimental CD analysis (Figure 5c,d). Tm values of CCCT6 and CCCTA2 were 316 K and 329 K, respectively (Figure 5b). Therefore, these results confirmed that the estimated Tm value of CCCT6 is well matched to the experimentally determined Tm. In the case of CCCTA2, although the Tm value was not exactly predicted, the estimation is very close to the actual value. These results suggest that the TR plot is useful to predict or estimate the Tm value that represents the structural stability of the i-motif.

### 2.7. MD Simulation Based Thermal Stability Prediction of I-Motif (C4): MD-TSPC4

**Figure 6:**
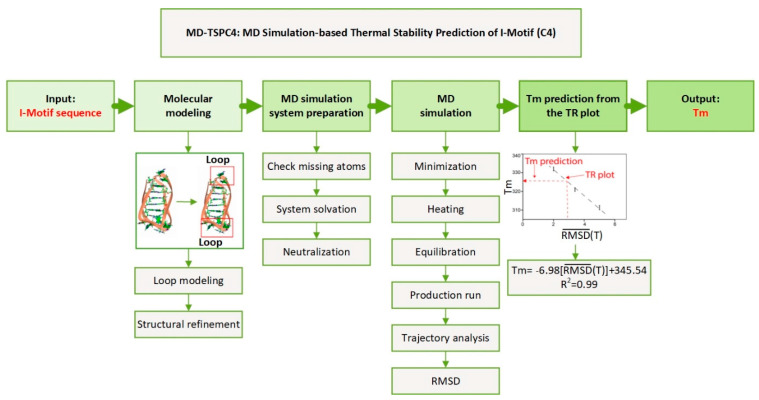
Schematic illustration of MD simulation-based Thermal Stability Prediction of i-motif (C4) (MD-TSPC4). The stepwise methodology explains how Tm value is predicted from the input i-motif sequence: model building by molecular modeling, preparation of MD simulation, MD simulation at five temperatures, and Tm prediction from $\bar{RMSD}\left(T\right)$ by applying the TR plot.

We found the correlation between RMSD obtained by the computational method and experimentally determined Tm value and further proved that the TR plot can be used for estimating the Tm value by MD simulation. Based on this finding, we propose a new computation method for the thermal stability prediction of i-motifs (C4) based on the MD simulation, and thus named it as MD-TSPC4 (Figure 6). For each new i-motif sequence, model building, MD simulation using AMBER program and Tm prediction from the TR plot can be sequentially performed. When we applied this method for predicting the Tm values of CCCT6 or CCCTA2 using the computer server Intel(R) Xeon(R) CPU E5-2640 v4 (2.40GHz) 10 core processor with four GPUs, we can finish the calculation within three days. We expect that the Tm value of any i-motif can be estimated within hours when the high-performance server is used, without performing real experiments in which ODN synthesis and CD measurement are necessary Therefore, we expect that this method can be useful for providing key information when studying a novel i-motif and genome wide or high throughput characterization of i-motifs.

## 3. Discussion

The i-motif forms a stable structure transiently in cells under specific conditions and is involved in various key cellular processes including transcription regulation [31]. Therefore, it is expected that the structural stability of i-motifs is highly correlated with their functionality. Accordingly, estimating the structural stability of any functional i-motif is important to design and perform experiments required for the elucidating the biological implication of i-motifs. However, there are limitations in performing experimental validation of the structural stability of i-motif. To overcome this issue, we developed a new computational method, MD-TSPC4, that is used for estimating the thermal or structural stability in terms of providing the Tm value (Figure 6).

MD-TSPC4 was generated based on the assumption that conformational flexibility that is interpreted by the $\bar{RMSD}\left(T\right)$ values for the MD simulation is directly correlated to the thermal stability in terms of the experimentally obtained Tm value. Under this assumption, we made a plot representing the correlation between Tm and $\bar{RMSD}\left(T\right)$ (TR plot), which was validated by predicting the Tm value of the independent model i-motif (CCCT6) and the well-characterized i-motif (CCCTA2) present in the human telomere (Figure 5). In this plot, we used the average RMSD of MD simulation conducted at low to high temperatures instead of RMSD of MD simulation performed at any temperature, since using the average of RMSDs obtained at the five different temperatures provides the plot with a better correlation coefficient. This result shows that the uncertainties in MD simulations or limitations during computation at one temperature can be reduced when results at various temperatures are considered and averaged. This assumption is more applicable for the long loop i-motifs of which conformational flexibility is not directly correlated to temperature (Figure 1 and Table 2). 

In this method, we applied model i-motifs with thymine bases but different loop lengths, since the effect of thymine on thermal stability has been well studied [41]. However, we validated that this method can be used for the prediction of the Tm value of i-motifs containing loops with a mixture of adenine and thymine by applying MD-TSPC4 (Figure 6) to predict the Tm of CCCTA2 (Figure 5). The base composition in the loop region is an important factor that affects the stability of the structure of an i-motif-forming sequence [40]. We expected that adding adenine instead of thymine would increase the structural flexibility due to the high degree of freedom in adenine compared to thymine. As expected, the experimental Tm value as well as the calculated $\bar{RMSD}\left(T\right)$ of CCCTA2 was lower than that of CCCT3 (Figure 5). As a result, the expected Tm value (331 K) closely matched the experimentally determined Tm (329 K). Therefore, we anticipate that this method can be applied to predict the Tm values of any novel i-motifs with various loop length and composition. 

Since this prediction method was made using a limited number of model i-motifs, it might be not suitable for some i-motif sequences. However, since we have proven the correlation between the thermal stability and conformational flexibility, we believe that the TR plot based MD-TSPC4 method can be further optimized and refined by including more experimental and MD simulation results, and thus more accurate prediction can be achievable. In addition, we anticipate that this predicting method can be further developed for estimating effects of other factors such as pH on the i-motif stability. From computational and experimental approaches, 264,664 and 716,310 G4s have been identified in the human genome [42], suggesting that a similar number of i-motifs may be present in the human genome, since i-motifs can be present in the complementary positions to the G4 sites. Although fewer studies have examined i-motifs compared with G4s, recent evidence suggests that i-motifs are present in live cells and actively involved in various cellular processes. Therefore, new tools to study i-motifs in terms of their biophysical and cellular properties are urgently required. From this point of view, we believe that MD-TSC4 can contribute to the advancement of i-motif study and nucleic acid biology.

## 4. Materials and Methods 

### 4.1. Model I-Motif Sequences

For the modeling and experimental studies, we designed five i-motif models with various loop lengths and sequences (Table 1). 

### 4.2. Molecular Modeling

The crystal structure (PDB ID: 1ELN) of the human telomeric i-motif was used as a template for modeling. The loop was modeled using WinCoot [43]. Energy minimization of the models was performed by UCSF Chimera [44]. 

### 4.3. MD Simulations

MD simulation has been performed in explicit solvent using the AMBER package (version 16) [45]. The BSC1 force field [46] was used to describe DNA force field parameters for cytosine protonation of the i-motif structure [47]. Each i-motif system was prepared using the tleap module of AMBER 16 by soaking the system in 8 Å layer of TIP3P water molecules in the presence of sodium counter ions, depending on the number of protonated cytosines. MD simulation was performed in the NTP ensemble using the SHAKE algorithm [48] to constrain covalent bonds involving hydrogens, with periodic boundary conditions and temperature. The i-motif system was minimized using a two-step approach. In the first step, we kept the DNA fixed and minimized the positions of the water and ions. In the second step, we minimized the entire i-motif system in the presence of solvent and ions. We preprocessed the systems with external heat and volume scaling at 0.2 ps to keep the system stable at 300 K and 1 atm. During the MD simulation, trajectory files were saved every 0.5 picoseconds (ps). The Pmemd.cuda was used to obtain the MD simulation trajectories while analyses were done by PTRAJ/CPPTRAJ module [49] and xmgrace [50]. 

### 4.4. Trajectory Analysis

In post-simulation analysis of trajectories, the CPPTRAJ module was used to analyze the root mean square deviation (RMSD), root mean square fluctuation (RMSF) and radius distribution function (RDF) [51,52]. The RMSD values were calculated for the whole i-motif, core region and loop region, respectively, using the starting structure as a reference frame and the deviation of the coordinate of given set of atoms in a time interval. The RMSF was calculated to measure the fluctuations of each residue from its mean position. The protonated (C:C+) trajectory was analyzed by radial distribution function to evaluate the base density variation as a function of distance (R) from a reference point. Distance (R) between protonated hydrogen and CN3 was measured in C:C+ base pairs and atom densities are calculated. The RDF of atoms was normalized by the expected number of particles at that distance. The normalization was calculated as follows:

$$Density*(\left\{{\frac{4\pi }{3\left(R+dR\right)}}^{3}\right\}-\{4\pi /3{\left(dR\right)}^{3}\})$$

where dR is equal to the bin spacing. The default density value is 0.03 molecules Å^−3^, which corresponds to a density of water approximately equal to 1.0 gmL^−1^.

### 4.5. Per Residue Binding Free Energy Calculation

Molecular Mechanics Generalized Born Surface Area (MMGBSA) [53,54] method implemented in the AMBER package 16 [45] was used for calculating the binding free energy of each residue. For the Generalized Born (GB) calculation, the set of charge derived by Massova and Kollman (1999) was used [55]. 

### 4.6. Oligonucleotide Preparation

The oligodeoxynucleotides were synthesized by Bionics (Korea) for the circular dichroism (CD) experiment. They were suspended in 20 mM sodium cacodylate buffer (pH 5.0) to a working concentration of 15.0 µM. Annealing was done by heating at 95 °C for 5 min and slowly cooling to room temperature for 5 h. The oligos were stored at 4 °C for overnight incubation before the experiment. 

### 4.7. CD Spectroscopy

CD spectra were measured at 20 °C between 220 and 320 nm wavelength with a speed of 100 nm/min, 1 nm data pitch and bandwidth of 1 nm using a 1 mm quartz cuvette with 200 µL reaction volume. CD experiments were performed on a Jasco J-810 CD spectropolarimeter fitted with a Jasco CDC-426F Peltier temperature controller. For thermal melting analysis, the samples were heated from 20–80 °C (293–353 K) with a gradient 2 °C/min and data pitch 0.2, the annealing curve was also obtained. The ellipticity was recorded at 285 nm. Data plotting and curve fitting were performed on GraphPad Prism 8.4.2. The melting temperature (T_m_) of model i-motifs at each condition was calculated from the 1st derivative of CD ellipticity at 285 nm vs. temperature. Final Tm value at each condition was obtained after averaging the results of 3 individual melting experiments.

## Figures and Tables

**Table 1:** Model i-motifs used in this study and their sequences.

Name	Sequence (5′-3′)	** Model
CCCT3	* d[(CCCTTT)_3_CCC]	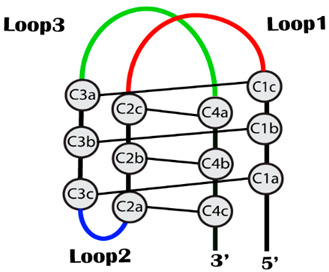
CCCT5	d[(CCCTTTTT)_3_CCC]	
CCCT6	d[(CCCTTTTTT)_3_CCC]	
CCCT7	d[(CCCTTTTTTT)_3_CCC]	
CCCTA2	d[(CCCTAA)_3_CCC]	

* ‘d’ in front of sequence represents the “deoxy”. ** Model shows the overall structure and nomenclature of the core and loop regions of the model i-motifs used in this study.

**Table 2:** Average RMSD of atoms in the whole i-motif, core and loop regions during 200 ns simulation at five different temperatures for model i-motifs (CCCT3, CCCT5, and CCCT7) used for making the TR plot and model i-motifs used for validation (CCCT6, and CCCTA2).

Model I-Motifs															
RMSD (Å)															
Temperature	CCCT3			CCCT5			CCCT7			CCCT6			CCCTA2		
	Whole I-Motif	Core Region	Loop Region	Whole I-Motif	Core Region	Loop Region	Whole I-Motif	Core Region	Loop Region	Whole I-Motif	Core Region	Loop Region	Whole I-Motif	Core Region	Loop Region
280 K	1.8	1.2	3.5	3.8	1.2	6.1	5.3	1.5	7.0	3.5	1.5	5.3	1.9	1.4	4.0
300 K	1.9	1.3	4.2	3.3	1.1	4.9	4.6	2.6	6.2	3.4	0.9	5.4	1.9	1.3	4.3
320 K	2.1	1.2	4.4	3.0	1.3	5.6	4.1	1.9	5.9	4.3	1.0	5.9	2.1	1.3	4.4
340 K	2.1	1.2	4.5	3.1	1.0	5.9	5.4	1.3	8.7	4.6	2.3	7.2	2.5	1.3	4.8
360 K	2.4	1.2	4.5	4.0	1.2	6.4	5.8	1.4	7.7	4.8	1.1	7.1	2.2	1.5	5.1
$\bar{RMSD}\;\left(T\right)$	2.0	1.2	4.2	3.4	1.2	5.8	5.0	1.7	7.1	4.1	1.3	6.2	2.1	1.4	4.5

## Data Availability

Data sharing not applicable.

## Supplementary Materials

The following are available online at https://www.mdpi.com/1422-0067/22/1/61/s1, Figure S1: Model structures of i-motifs, CCCT3, CCCT5 and CCCT7, after 200 ns molecular dynamics simulation at 280 K. The backbone and bases are represented as ribbon and stick diagrams, respectively. Bases in the core regions are drawn in green, while those in loop1, 2 and 3 are presented in yellow, magenta and blue, respectively. Stacking interactions of whole i-motif structures are shown on the left while interactions between loop1 and loop3 are illustrated on the right. Figure S2: Radial distribution functions [g(r)] for hydrogen bonds in C:C+ base pairs of the three model i-motif structures collected at the initial (1 ns) and final (200 ns) time steps during the MD simulation. Radial distribution functions at different temperatures are displayed in different colors. The color schemes are explained in the insets (bottom right). The area near the peak of the radial distribution functions is enlarged for clarification and shown in the insets (top right). Figure S3: Graphical representation of the root means square deviation (RMSD) of whole atoms in i-motif during 200 ns molecular dynamic simulation. RMSD changes in the deprotonated and protonated CCCT3 i-motifs are displayed in black and red, respectively. Figure S4: The melting temperature (Tm) and annealing temperature (Ta) of each i-motif sample are calculated from their CD melting and annealing graphs. CD melting and annealing curves were obtained at 285 nm wavelength within temperature range (293-353 K). Table S1: Hydrogen bond distances in C:C+ of model i-motif structures.

## References

[1] Sen D., Gilbert W. (1988). Formation of parallel four-stranded complexes by guanine-rich motifs in DNA and its implications for meiosis. Nat. Cell Biol..

[2] Gehring K., Leroy J.-L., Guéron M. (1993). A tetrameric DNA structure with protonated cytosine-cytosine base pairs. Nat. Cell Biol..

[3] Felsenfeld G., Rich A. (1957). Studies on the formation of two- and three-stranded polyribonucleotides. Biochim. Biophys. Acta (BBA) Bioenerg..

[4] Panayotatos N., Wells R.D. (1981). Cruciform structures in supercoiled DNA. Nature.

[5] Ravichandran S., Subramani V., Kim K.K. (2019). Z-DNA in the genome: From structure to disease. Biophys. Rev..

[6] Dai X., Greizerstein M.B., Nadas-Chinni K., Rothman-Denes L.B. (1997). Supercoil-induced extrusion of a regulatory DNA hairpin. Proc. Natl. Acad. Sci. USA.

[7] Thompson C.A.H., Wong J.M.Y. (2020). Non-canonical Functions of Telomerase Reverse Transcriptase: Emerging Roles and Biological Relevance. Curr. Top. Med. Chem..

[8] Tateishi-Karimata H., Sugimoto N. (2020). Chemical biology of non-canonical structures of nucleic acids for therapeutic applications. Chem. Commun..

[9] Huppert J.L., Bugaut A., Kumari S., Balasubramanian S. (2008). G-quadruplexes: The beginning and end of UTRs. Nucleic Acids Res..

[10] Beaudoin J.D., Perreault J.P. (2010). 5’-UTR G-quadruplex structures acting as translational repressors. Nucleic Acids Res..

[11] Endoh T., Kawasaki Y., Sugimoto N. (2013). Suppression of gene expression by G-quadruplexes in open reading frames depends on G-quadruplex stability. Angew. Chem. Int. Ed Engl..

[12] Huppert J.L., Balasubramanian S. (2005). Prevalence of quadruplexes in the human genome. Nucleic Acids Res..

[13] Brooks T.A., Hurley L.H. (2010). Targeting MYC Expression through G-Quadruplexes. Genes Cancer.

[14] Guo K., Gokhale V., Hurley L.H., Sun D. (2008). Intramolecularly folded G-quadruplex and i-motif structures in the proximal promoter of the vascular endothelial growth factor gene. Nucleic Acids Res..

[15] Sengar A., Vandana J.J., Chambers V.S., Di Antonio M., Winnerdy F.R., Balasubramanian S., Phan A.T. (2019). Structure of a (3+1) hybrid G-quadruplex in the PARP1 promoter. Nucleic Acids Res..

[16] Amato J., Pagano A., Capasso D., Di Gaetano S., Giustiniano M., Novellino E., Randazzo A., Pagano B. (2018). Targeting the BCL2 Gene Promoter G-Quadruplex with a New Class of Furopyridazinone-Based Molecules. ChemMedChem.

[17] Cogoi S., Xodo L.E. (2006). G-quadruplex formation within the promoter of the KRAS proto-oncogene and its effect on transcription. Nucleic Acids Res..

[18] Garg R., Aggarwal J., Thakkar B. (2016). Genome-wide discovery of G-quadruplex forming sequences and their functional relevance in plants. Sci. Rep. UK.

[19] Ravichandran S., Ahn J.H., Kim K.K. (2019). Unraveling the Regulatory G-Quadruplex Puzzle: Lessons From Genome and Transcriptome-Wide Studies. Front. Genet.

[20] Ravichandran S., Kim Y.E., Bansal V., Ghosh A., Hur J., Subramani V.K., Pradhan S., Lee M.K., Kim K.K., Ahn J.H. (2018). Genome-wide analysis of regulatory G-quadruplexes affecting gene expression in human cytomegalovirus. PLoS Pathog..

[21] Parveen N., Shamim A., Cho S., Kim K.K. (2019). Computational Approaches to Predict the Non-canonical DNAs. Curr. Bioinform..

[22] Lombardi E.P., Londono-Vallejo A. (2020). A guide to computational methods for G-quadruplex prediction. Nucleic Acids Res..

[23] Marsico G., Chambers V.S., Sahakyan A.B., McCauley P., Boutell J.M., Antonio M.D., Balasubramanian S. (2019). Whole genome experimental maps of DNA G-quadruplexes in multiple species. Nucleic Acids Res..

[24] Dhakal S., Yu Z., Konik R., Cui Y., Koirala D., Mao H. (2012). G-quadruplex and i-motif are mutually exclusive in ILPR double-stranded DNA. Biophys. J..

[25] Kang H.J., Kendrick S., Hecht S.M., Hurley L.H. (2014). The transcriptional complex between the BCL2 i-motif and hnRNP LL is a molecular switch for control of gene expression that can be modulated by small molecules. J. Am. Chem. Soc..

[26] Niu K., Zhang X., Deng H., Wu F., Ren Y., Xiang H., Zheng S., Liu L., Huang L., Zeng B. (2018). BmILF and i-motif structure are involved in transcriptional regulation of BmPOUM2 in Bombyx mori. Nucleic Acids Res..

[27] Khan N., Avino A., Tauler R., Gonzalez C., Eritja R., Gargallo R. (2007). Solution equilibria of the i-motif-forming region upstream of the B-cell lymphoma-2 P1 promoter. Biochimie.

[28] Bucek P., Jaumot J., Avino A., Eritja R., Gargallo R. (2009). pH-Modulated Watson-Crick duplex-quadruplex equilibria of guanine-rich and cytosine-rich DNA sequences 140 base pairs upstream of the c-kit transcription initiation site. Chemistry.

[29] Sun D., Hurley L.H. (2009). The importance of negative superhelicity in inducing the formation of G-quadruplex and i-motif structures in the c-Myc promoter: Implications for drug targeting and control of gene expression. J. Med. Chem..

[30] Guo K., Pourpak A., Beetz-Rogers K., Gokhale V., Sun D., Hurley L.H. (2007). Formation of pseudosymmetrical G-quadruplex and i-motif structures in the proximal promoter region of the RET oncogene. J. Am. Chem. Soc..

[31] Zeraati M., Langley D.B., Schofield P., Moye A.L., Rouet R., Hughes W.E., Bryan T.M., Dinger M.E., Christ D. (2018). I-motif DNA structures are formed in the nuclei of human cells. Nat. Chem..

[32] Abou Assi H., Garavis M., Gonzalez C., Damha M.J. (2018). i-Motif DNA: Structural features and significance to cell biology. Nucleic Acids Res..

[33] McKim M., Buxton A., Johnson C., Metz A., Sheardy R.D. (2016). Loop Sequence Context Influences the Formation and Stability of the i-Motif for DNA Oligomers of Sequence (CCCXXX)4, where X = A and/or T, under Slightly Acidic Conditions. J. Phys. Chem. B.

[34] Reilly S.M., Morgan R.K., Brooks T.A., Wadkins R.M. (2015). Effect of interior loop length on the thermal stability and pK(a) of i-motif DNA. Biochemistry.

[35] Bhavsar-Jog Y.P., Van Dornshuld E., Brooks T.A., Tschumper G.S., Wadkins R.M. (2014). Epigenetic Modification, Dehydration, and Molecular Crowding Effects on the Thermodynamics of i-Motif Structure Formation from C-Rich DNA. Biochemistry.

[36] Kaiser C.E., Van Ert N.A., Agrawal P., Chawla R., Yang D., Hurley L.H. (2017). Insight into the Complexity of the i-Motif and G-Quadruplex DNA Structures Formed in the KRAS Promoter and Subsequent Drug-Induced Gene Repression. J. Am. Chem. Soc..

[37] Benabou S., Garavis M., Lyonnais S., Eritja R., Gonzalez C., Gargallo R. (2016). Understanding the effect of the nature of the nucleobase in the loops on the stability of the i-motif structure. Phys. Chem. Chem. Phys..

[38] Mir B., Serrano I., Buitrago D., Orozco M., Escaja N., Gonzalez C. (2017). Prevalent Sequences in the Human Genome Can Form Mini i-Motif Structures at Physiological pH. J. Am. Chem. Soc..

[39] Phan A.T., Gueron M., Leroy J.L. (2000). The solution structure and internal motions of a fragment of the cytidine-rich strand of the human telomere. J. Mol. Biol..

[40] Gurung S.P., Schwarz C., Hall J.P., Cardin C.J., Brazier J.A. (2015). The importance of loop length on the stability of i-motif structures. Chem. Commun..

[41] Pages B.J., Gurung S.P., McQuaid K., Hall J.P., Cardin C.J., Brazier J.A. (2019). Stabilization of Long-Looped i-Motif DNA by Polypyridyl Ruthenium Complexes. Front. Chem..

[42] Sahakyan A.B., Chambers V.S., Marsico G., Santner T., Di Antonio M., Balasubramanian S. (2017). Machine learning model for sequence-driven DNA G-quadruplex formation. Sci. Rep..

[43] Emsley P., Lohkamp B., Scott W.G., Cowtan K. (2010). Features and development of Coot. Acta Crystallogr. Sect. D Biol. Crystallogr..

[44] Pettersen E.F., Goddard T.D., Huang C.C., Couch G.S., Greenblatt D.M., Meng E.C., Ferrin T.E. (2004). UCSF Chimera--a visualization system for exploratory research and analysis. J. Comput. Chem..

[45] Case D.A., Betz R.M., Cerutti D.S., Cheatham T.E., Darden T.A., Duke R.E., Giese T.J., Gohlke H., Goetz A.W., Homeyer N. (2016). AMBER 2016.

[46] Ivani I., Dans P.D., Noy A., Perez A., Faustino I., Hospital A., Walther J., Andrio P., Goni R., Balaceanu A. (2016). Parmbsc1: A refined force field for DNA simulations. Nat. Methods.

[47] Sponer J., Burda J.V., Mejzlik P., Leszczynski J., Hobza P. (1997). Hydrogen-bonded trimers of DNA bases and their interaction with metal cations: Ab initio quantum-chemical and empirical potential study. J. Biomol. Struct. Dyn..

[48] Ryckaert J.P., Ciccotti G., Berendsen H.J. (1977). Numerical integration of the cartesian equations of motion of a system with constraints: Molecular dynamics of n-alkanes. J. Comput. Phys..

[49] Roe D.R., Cheatham T.E. (2013). PTRAJ and CPPTRAJ: Software for Processing and Analysis of Molecular Dynamics Trajectory Data. J. Chem. Theory Comput..

[50] Vaught A. (1996). Graphing with Gnuplot and Xmgr: Two graphing packages available under linux. Linux J..

[51] Zar J.H. (1999). Biostatistical Analysis.

[52] Box G.H.W., Hunter J.S. (1978). Statistics for Experimenters: An Introduction to Design, Data Analysis, and Model Building.

[53] Tsui V., Case D.A. (2000). Molecular dynamics simulations of nucleic acids with a generalized Born solvation model. J. Am. Chem. Soc..

[54] Kollman P.A., Massova I., Reyes C., Kuhn B., Huo S., Chong L., Lee M., Lee T., Duan Y., Wang W. (2000). Calculating structures and free energies of complex molecules: Combining molecular mechanics and continuum models. Accounts Chem. Res..

[55] Massova I., Kollman P.A. (2000). Combined molecular mechanical and continuum solvent approach (MM-PBSA/GBSA) to predict ligand binding. Perspect. Drug Discov. Des..

