# Supplementary material for: MD-TSPC4: Computational Method for Predicting the Thermal Stability of I-Motif
Source: Int J Mol Sci. 2020 Dec 23;22(1):61. doi: 10.3390/ijms22010061 (PMC7793491; doi:10.3390/ijms22010061)
Supplement: Supplementary file 1 [file ijms-22-00061-s001.pdf]

Supplementary Data

MD-TSPC4: Computational method for predicting the thermal stability of i-motif

Amen Shamim[1,2], Maria Razzaq[1], Kyeong Kyu Kim[1*]

[1] Department of Precision Medicine, Sungkyunkwan University School of Medicine, Suwon, 16419, Republic of Korea
[2] Center of Agricultural Biochemistry and Biotechnology (CABB), University of Agriculture, Faisalabad, 38040, Pakistan
* Correspondence: kyeongkyu@skku.edu (K.K.K), Tel: 82-31-299-6136, Fax: 82-31-299-6159

Received: 24 November 2020; Accepted: 17 December 2020; Published: 23 December 2020

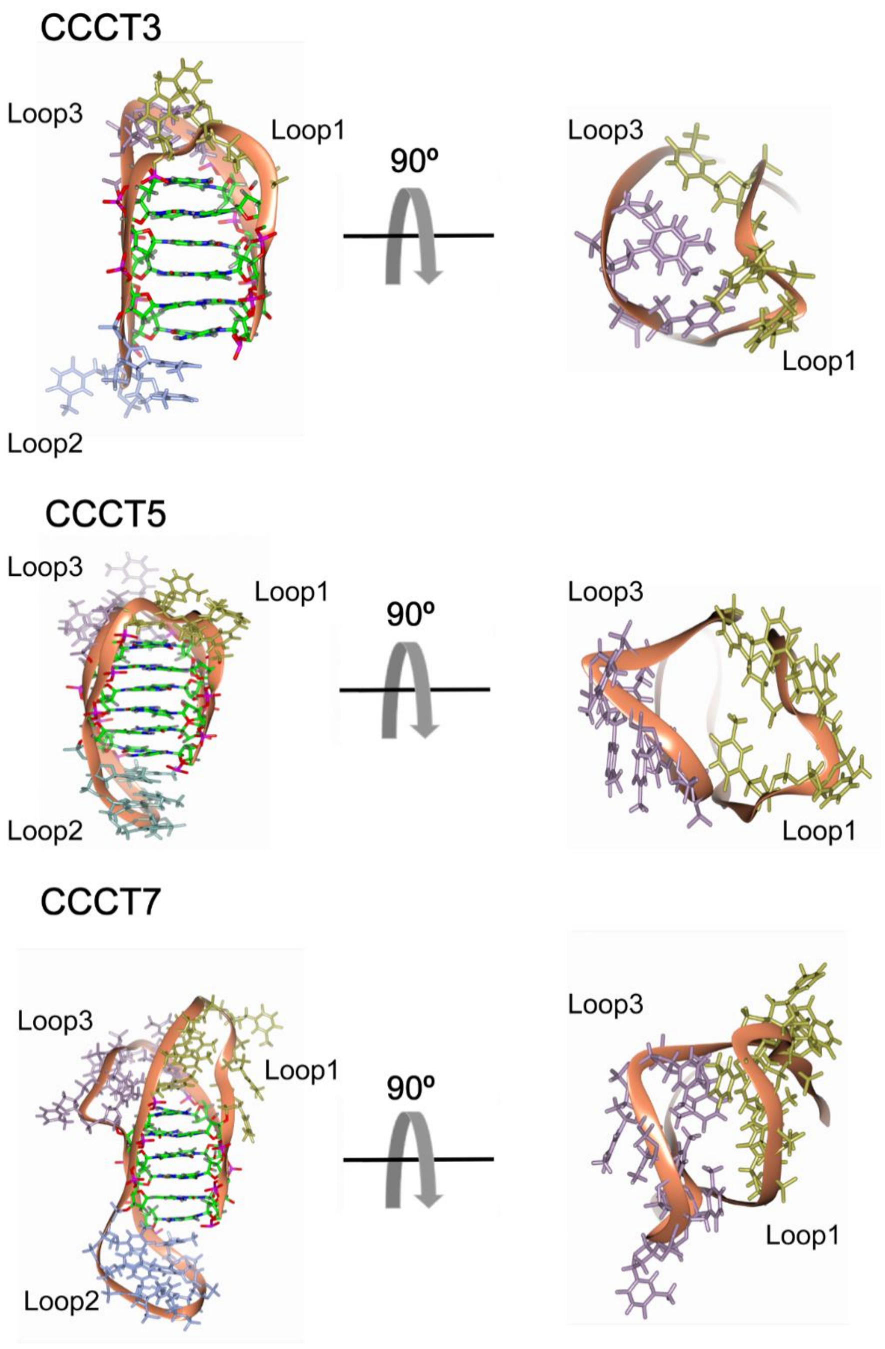

Figure S1: Model structures of i-motifs, CCCT3, CCCT5 and CCCT7, after 200 ns molecular dynamics simulation at 280 K. The backbone and bases are represented as ribbon and stick diagrams, respectively. Bases in the core regions are drawn in green, while those in loop1, 2 and 3 are presented in yellow, magenta and blue, respectively. Stacking interactions of whole i-motif structures are shown on the left while interactions between loop1 and loop3 are illustrated on the right.

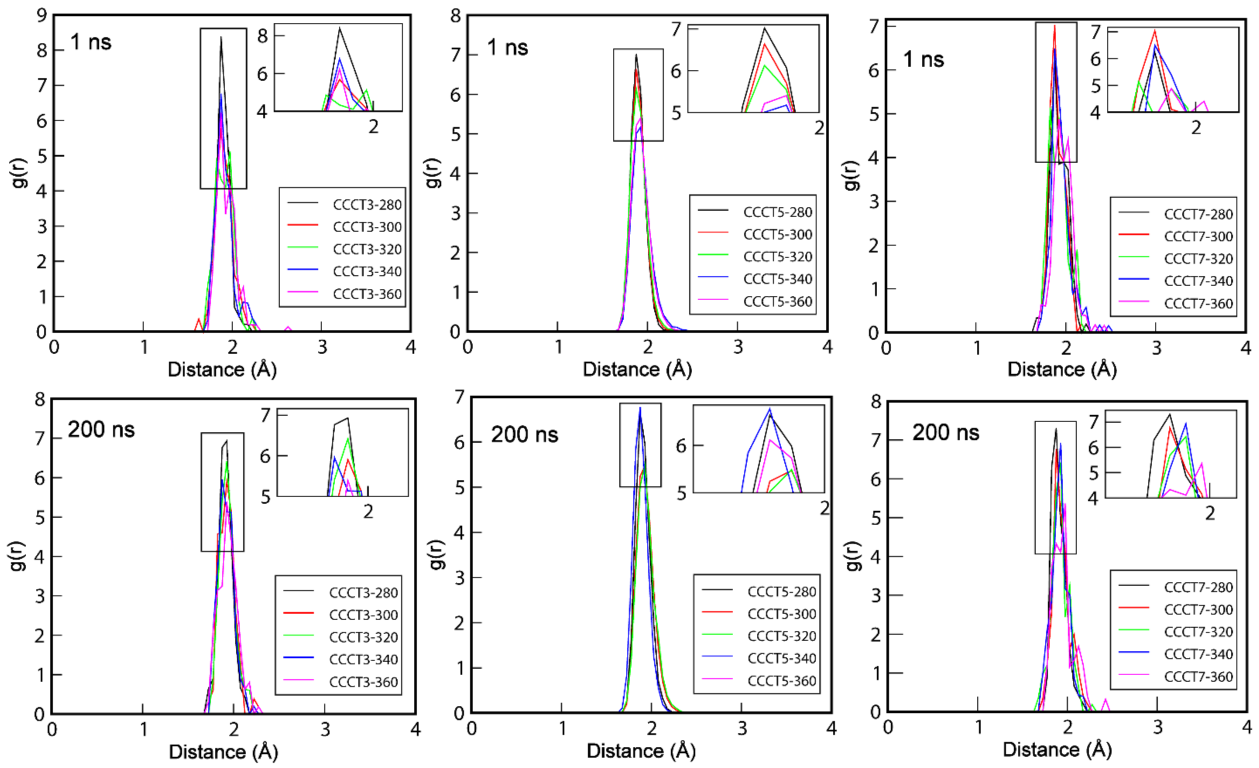

Figure S2: Radial distribution functions [g(r)] for hydrogen bonds in C:C$^+$ base pairs of the three model i-motif structures collected at the initial (1 ns) and final (200 ns) time steps during the MD simulation. Radial distribution functions at different temperatures are displayed in different colors. The color schemes are explained in the insets (bottom right). The area near the peak of the radial distribution functions is enlarged for clarification and shown in the insets (top right).

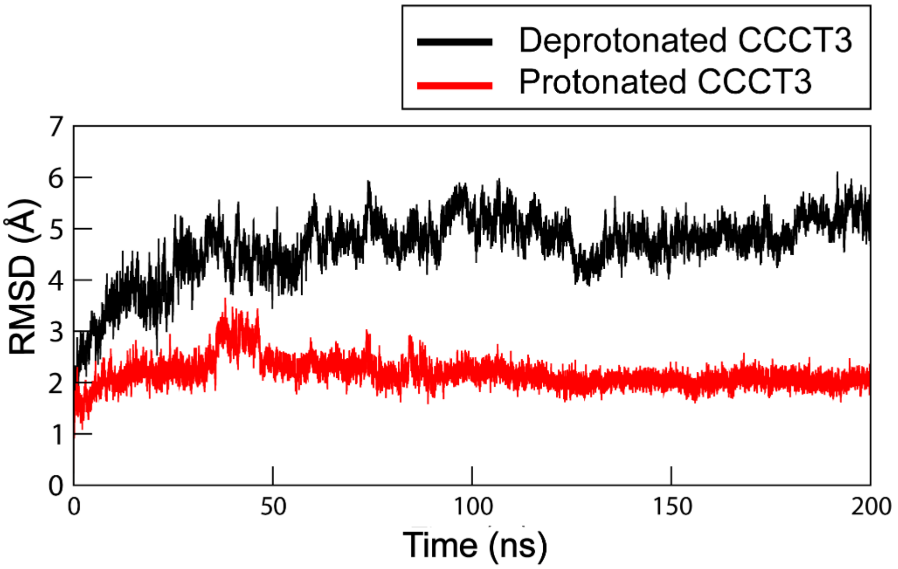

Figure S3: Graphical representation of the root means square deviation (RMSD) of whole atoms in i-motif during 200 ns molecular dynamic simulation. RMSD changes in the deprotonated and protonated CCCT3 i-motifs are displayed in black and red, respectively.

Table S1: Hydrogen bond distances in C:C+ of model i-motif structures.

**Models***	**Atoms**	**N3-H+(Å)**	**Models**	**Atoms**	**N3-H+(Å)**	**Models**	**Atoms**	**N3-H+(Å)**
CCCT3-280	C1a-C3c	1.9	CCCT5-280	C1a-C3c	1.9	CCCT7-280	C1a-C3c	1.8
	C1b-C3b	1.9		C1b-C3b	1.9		C1b-C3b	2.0
	C1c-C3a	2.1		C1c-C3a	2.1		C1c-C3a	1.9
	C2a-C4c	2.0		C2a-C4c	2.0		C2a-C4c	2.0
	C2b-C4b	1.9		C2b-C4b	1.9		C2b-C4b	1.9
	C2c-C4a	1.9		C2c-C4a	1.9		C2c-C4a	2.0
CCCT3-300	C1a-C3c	1.9	CCCT5-300	C1a-C3c	1.9	CCCT7-300	C1a-C3c	1.9
	C1b-C3b	1.9		C1b-C3b	1.9		C1b-C3b	2.0
	C1c-C3a	1.8		C1c-C3a	1.8		C1c-C3a	2.1
	C2a-C4c	1.7		C2a-C4c	1.7		C2a-C4c	1.9
	C2b-C4b	1.9		C2b-C4b	1.9		C2b-C4b	1.9
	C2c-C4a	1.9		C2c-C4a	1.9		C2c-C4a	10.4
CCCT3-320	C1a-C3c	2.0	CCCT5-320	C1a-C3c	2.0	CCCT7-320	C1a-C3c	2.0
	C1b-C3b	1.9		C1b-C3b	1.9		C1b-C3b	2.0
	C1c-C3a	1.9		C1c-C3a	1.9		C1c-C3a	1.9
	C2a-C4c	1.9		C2a-C4c	1.9		C2a-C4c	2.2
	C2b-C4b	1.9		C2b-C4b	1.9		C2b-C4b	1.8
	C2c-C4a	1.9		C2c-C4a	1.9		C2c-C4a	9.9
	C2c-C4a	1.9		C2c-C4a	1.9		C2c-C4a	1.9
CCCT3-340	C1a-C3c	2.0	CCCT5-340	C1a-C3c	2.0	CCCT7-340	C1a-C3c	2.0
	C1b-C3b	1.9		C1b-C3b	1.9		C1b-C3b	2.1
	C1c-C3a	1.9		C1c-C3a	1.9		C1c-C3a	1.9
	C2a-C4c	1.9		C2a-C4c	1.9		C2a-C4c	2.0
	C2b-C4b	1.9		C2b-C4b	1.9		C2b-C4b	2.0
	C2c-C4a	1.9		C2c-C4a	1.9		C2c-C4a	2.1
CCCT3-360	C1a-C3c	2.0	CCCT5-360	C1a-C3c	2.0	CCCT7-360	C1a-C3c	1.9
	C1b-C3b	1.9		C1b-C3b	1.9		C1b-C3b	1.9
	C1c-C3a	1.9		C1c-C3a	1.9		C1c-C3a	2.0
	C2a-C4c	2.0		C2a-C4c	2.0		C2a-C4c	1.9
	C2b-C4b	2.0		C2b-C4b	2.0		C2b-C4b	1.9
	C2c-C4a	1.9		C2c-C4a	1.8		C2c-C4a	1.8

* Nomenclature of models: name of model i-motif - MD simulation temperature. For example, CCCT3-280 represents the model structure of CCCT3 at 200 ns time step with MD simulation at 280K
